# An eHealth Intervention to Improve Quality of Life, Socioemotional, and Health-Related Measures Among Older Adults With Multiple Chronic Conditions: Randomized Controlled Trial

**DOI:** 10.2196/59588

**Published:** 2024-12-06

**Authors:** David H Gustafson Sr, Marie-Louise Mares, Darcie Johnston, Olivia J Vjorn, John J Curtin, Gina Landucci, Klaren Pe-Romashko, David H Gustafson Jr, Dhavan V Shah

**Affiliations:** 1 Center for Health Enhancement Systems Studies University of Wisconsin–Madison Madison, WI United States; 2 Department of Industrial and Systems Engineering University of Wisconsin–Madison Madison, WI United States; 3 Department of Communication Arts University of Wisconsin–Madison Madison, WI United States; 4 Department of Psychology University of Wisconsin–Madison Madison, WI United States; 5 School of Journalism and Mass Communication University of Wisconsin–Madison Madison, WI United States

**Keywords:** eHealth, telemedicine, aged, geriatrics, multiple chronic conditions, social support, quality of life, primary care, mobile phone, smartphone

## Abstract

**Background:**

In the United States, over 60% of adults aged 65 years or older have multiple chronic health conditions, with consequences that include reduced quality of life, increasingly complex but less person-centered treatment, and higher health care costs. A previous trial of ElderTree, an eHealth intervention for older adults, found socioemotional benefits for those with high rates of primary care use.

**Objective:**

This study tested the effectiveness of an ElderTree intervention designed specifically for older patients with multiple chronic conditions to determine whether combining it with primary care improved socioemotional and physical outcomes.

**Methods:**

In a nonblinded randomized controlled trial, 346 participants recruited from primary care clinics were assigned 1:1 to the ElderTree intervention or an attention control and were followed for 12 months. All participants were aged 65 years or older and had electronic health record diagnoses of at least three of 11 chronic conditions. Primary outcomes were mental and physical quality of life, psychological well-being (feelings of competence, connectedness, meaningfulness, and optimism), and loneliness. Tested mediators of the effects of the study arm (ElderTree vs active control) on changes in primary outcomes over time were 6-month changes in health coping, motivation, feelings of relatedness, depression, and anxiety. Tested moderators were sex, scheduled health care use, and number of chronic conditions. Data sources were surveys at baseline and 6 and 12 months comprising validated scales, and continuously collected ElderTree usage.

**Results:**

At 12 months, 76.1% (134/176) of ElderTree participants were still using the intervention. There was a significant effect of ElderTree (vs control) on improvements over 12 months in mental quality of life (arm × timepoint interaction: b=0.76, 95% CI 0.14-1.37; *P*=.02; 12-month ∆d=0.15) but no such effect on the other primary outcomes of physical quality of life, psychological well-being, or loneliness. Sex moderated the effects of the study arm over time on mental quality of life (b=1.33, 95% CI 0.09-2.58; *P*=.04) and psychological well-being (b=1.13, 95% CI 0.13-2.12; *P*=.03), with stronger effects for women than men. The effect of the study arm on mental quality of life was mediated by 6-month improvements in relatedness (α=1.25, *P*=.04; *b*=0.31, *P*<.001). Analyses of secondary and exploratory outcomes showed minimal effects of ElderTree.

**Conclusions:**

Consistent with the previous iteration of ElderTree, the current iteration designed for older patients with multiple chronic conditions showed signs of improving socioemotional outcomes but no impact on physical outcomes. This may reflect the choice of chronic conditions for inclusion, which need not have impinged on patients’ physical quality of life. Two ongoing trials are testing more specific versions of ElderTree targeting older patients coping with (1) chronic pain and (2) greater debilitation owing to at least 5 chronic conditions.

**Trial Registration:**

ClinicalTrials.gov NCT03387735; https://clinicaltrials.gov/study/NCT03387735

**International Registered Report Identifier (IRRID):**

RR2-10.2196/25175

## Introduction

### Background

Global data indicate that among adults with a chronic disease, more than half have multiple diagnosable conditions [1]. Although the consequences of multimorbidity vary by the particular array of conditions, research often indicates a compounding effect on the complexity of medical treatment [2-4], a reduction in patient-centered care as clinicians respond to the most pressing medical needs [5], and markedly increased medical costs [6-8]. For patients, multiple chronic conditions (MCCs) heighten the treatment burden [9] and the risk of adverse outcomes such as prolonged hospitalization and mortality [10]. Research also suggests that patients with MCCs tend to experience reduced quality of life (QOL) [11,12] as well as greater depression [13,14] and loneliness [15].

The prevalence of multimorbidity increases with age and low-income status. Assessments of United States data from 2018 found diagnoses of MCCs among more than 60% of those aged 65 years or older (vs 27.2% of the total adult population) and among 76.9% of older adults who qualified for low-income medical care (ie, “dual eligible” for Medicare and Medicaid) [16]. As such, the burden of MCCs often falls on those patients who may have fewer resources to navigate the complexities of their conditions.

One of the core challenges of MCCs is that the specific combination of conditions experienced by a given patient can vary widely. Clinical practice guidelines and recommendations for self-management tend to be based on specific diseases or conditions, and adjusting those guidelines and recommendations becomes more difficult as the number of comorbid conditions rises [17,18]. As various authors have noted, the risk of polypharmacy and the difficulty of self-management escalate with the number of comorbid conditions [19,20]. Given these challenges, the National Institutes of Health called for researchers to develop and test behavioral interventions that could be implemented in primary care and that would offer one broadly applicable and effective tool to help manage a wide array of combinations of chronic conditions [21]. This project was funded by the National Institutes of Health and responds to that call.

A recent review and meta-analysis of 16 randomized controlled trials (RCTs) of interventions to address multimorbidity in primary care and community settings was not encouraging [22]. Interventions were broadly grouped into those focused on medication management, self-management support, or care coordination plus self-management support. Most were designed to enhance patients’ interactions with their primary care team by coaching or informational sessions and materials. Across the 4753 participants, with most of them being older adults with 3 or more chronic conditions, there was “little or no evidence” of effects (relative to usual care [UC]) on health-related QOL or mental health, or on an array of secondary outcomes, including health care use, medication adherence, and self-efficacy. Notably, only 2 of the 16 interventions (both relatively brief, at 6 weeks in length) offered peer support in the form of weekly meetings [23,24], and none provided online resources for communicating with clinicians or peers.

A second meta-analysis, focusing on “digital telemedicine interventions” for patients with at least 2 comorbid chronic conditions [25], found somewhat more encouraging results, though the authors noted the prevalence of low-quality study designs, small sample sizes, and short durations (2-6 months with limited follow-up). The interventions were typically multifaceted, offering services such as telemonitoring (eg, online tracking of blood glucose, blood pressure, or weight, to be reviewed by the medical team), telecare (eg, online feedback or appointments with clinicians based on health-tracking scores and online yoga classes), or automated reminders (eg, for exercise or medications). The results across 2 or 3 studies for each outcome indicated moderate decreases in systolic blood pressure and cholesterol and small to moderate decreases in hemoglobin A_1c_. In contrast, the effects on patient-centered outcomes, such as QOL, perceived health status, and depression, were largely nonsignificant. Again, it was notable that no interventions involved peer support, and most focused on tracking specific health indicators rather than patient well-being.

The current RCT was designed to build on this prior work but to avoid key limitations, including small sample size, short intervention duration, and limited or no opportunity for communicating with clinicians or peers. It presents a relatively novel and rigorous approach to multimorbidity, testing a 12-month eHealth intervention designed to improve QOL, socioemotional outcomes, and health-related outcomes among older adults with 3 or more chronic conditions. The intervention, ElderTree (ET), is an information and support platform developed by our Center of Excellence in Active Aging, which was funded by the Agency for Healthcare Research and Quality, and is one of a collection of eHealth systems known as CHESS (Comprehensive Health Enhancement Support System) [26,27].

All CHESS systems, including ET, are built on the principles of continuing care and self-management, including long duration [28]; assertive outreach [29]; tracking [30]; prompts [31]; problem solving [32]; and peer, family, or clinical support [33]. In addition, CHESS systems are consistent with Self-Determination Theory, which asserts that satisfying the fundamental psychological needs of competence (feeling effective), relatedness (feeling connected to others), and autonomy (feeling internally motivated rather than coerced) contributes to adaptive functioning [34]. In randomized trials, our interventions significantly improved asthma control [35]; QOL and cost of care in HIV patients [36]; QOL and self-efficacy in breast cancer patients [37-39]; risky drinking [40]; and caregiver burden, symptom distress, and median length of survival in lung cancer patients [41].

ET was previously tested in an RCT involving 390 adults aged 65 years or older with at least one health risk factor (eg, recent fall, depression, or emergency room visit) in 3 Wisconsin communities (urban, suburban, and rural), who were followed for an intervention period of 12 months [27]. There were no significant effects in the sample as a whole, but subgroup analyses indicated that among participants who had 3 or more primary care visits in the 6 months prior to baseline, those in the ET group performed significantly better than those in the control group on measures of mental QOL, social support, and depression.

The results suggested that (1) ET may be more effective for patients who are dealing with multiple chronic conditions (MCCs), given that primary care use is relatively high among such patients, and (2) a system such as ET may be most effective if integrated into primary care. Building on those findings, this study examined the effects of an enhanced version of ET specifically for patients with MCCs rather than a general older population, focusing on not only QOL and socioemotional outcomes but also health measures and health care use.

### Study Objectives

The overall goal of the study was to assess the effects of ET versus attention control in older patients with MCCs. Our primary objective was to determine whether, and to what extent, supplementing patients’ UC with ET would lead to improvements in 4 primary outcomes. Like other interventions for chronic conditions (eg, those reviewed above), we assessed physical and mental QOL. Additionally, given the focus of our intervention on the core constructs of Self-Determination Theory (ie, motivation, competence, and autonomy), we assessed psychological well-being, using a measure that focused on feelings of competence, connectedness, meaningfulness, and optimism [42]. Finally, given the evidence about the bidirectional causal impact of loneliness on the progression of chronic conditions in older adults [43,44], we assessed participants’ loneliness. We also tested whether the effects of the study arm on these primary outcomes were mediated by 6-month changes in health-related coping strategies and motivation, social relatedness, anxiety, and depressive symptoms and whether effects were moderated by sex, scheduled health care use, and the number of chronic conditions.

As prespecified in the trial protocol [26], we tested the effects of the study arm (ET vs active control) on changes over time in a number of health-related secondary and exploratory outcomes: pain, number and severity of falls, symptom distress, medication adherence, use of crisis health care and long-term care, diet, alcohol use, cigarette use, and pain medication issues. To restrict the paper length, we only briefly summarize the results for these nonprimary outcomes. Complete descriptions and analyses of these variables are reported in Multimedia Appendix 1.

Lastly, the COVID-19 pandemic, which began mid-way through the study, overlapped with some portion of the 12-month intervention period for nearly half of the participants. As a result, we conducted post-hoc analyses, examining the months of overlap with the pandemic as a possible unanticipated moderator of the effects of the study arm.

## Methods

### Trial Design

**Figure 1:**
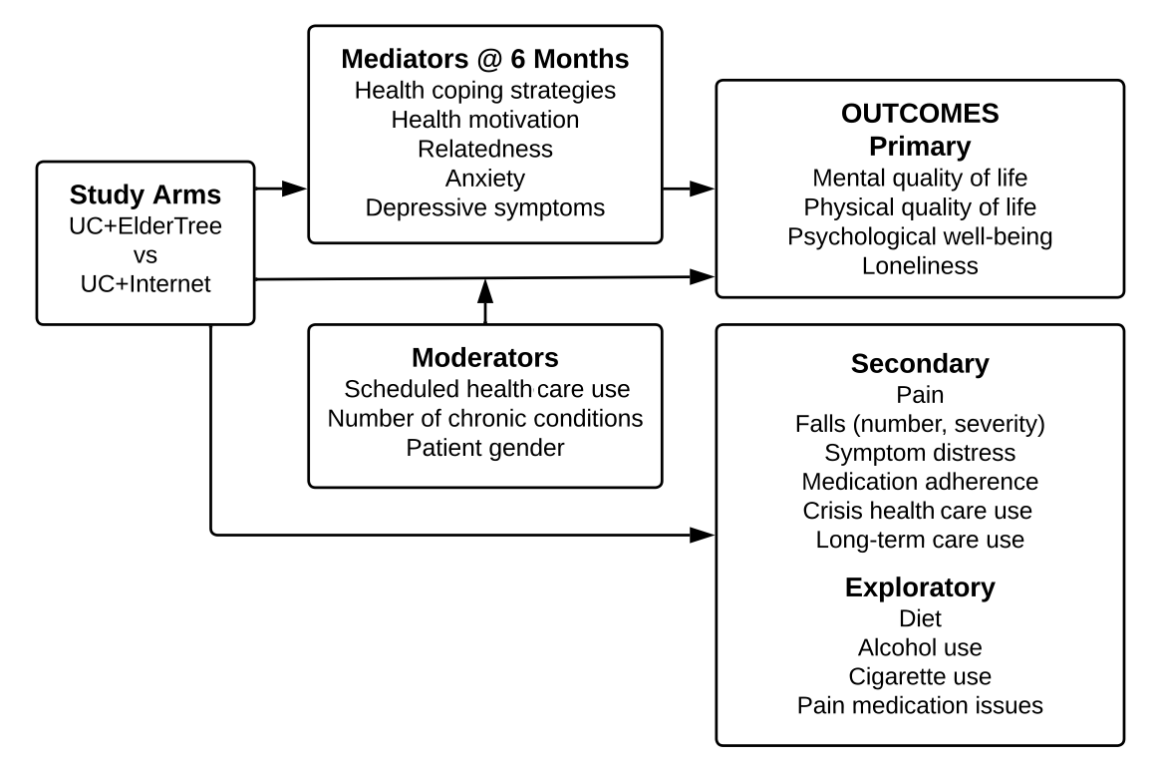
Study design. UC: usual care.

The trial was a nonblinded, randomized controlled design with 1:1 allocation to the intervention arm or an attention control for a period of 12 months. Assessments were conducted at baseline, 6 months, and 12 months. We originally planned an 18-month follow-up assessment (ie, 6 months after the end of the intervention). However, with the start of the pandemic and lock-down, as we considered the role that ET played in connecting participants to each other and to their primary care team, we (the study investigators) made an ethical decision to offer continued access to ET to those participants who were approaching months 13-18. This did not change the study design for the 12 months of the intervention, but it meant that the 18-month follow-up comparison between study arms was compromised: Participants in the ET arm varied in their access to the intervention during their final 6 months of the study, depending on whether that period coincided with the pandemic. We continued to gather the 18-month data to offer participants closure, but we no longer planned to analyze and did not reach out to participants in cases of incomplete or ambiguous data. As such, we did not analyze and do not report the 18-month results. Figure 1 shows the study design.

### Participants

Older adult patients with at least three chronic conditions were recruited from primary care clinics within the UW Health Department of Family Medicine and General Internal Medicine at the University of Wisconsin–Madison (UW Health). Eligible patients (1) were aged 65 years or older; (2) had been treated in the clinic for the previous 18 months or longer with no plans to leave during the study period; and (3) had diagnoses of 3 or more of the following 11 chronic conditions: hypertension, hyperlipidemia, diabetes, arthritis, BMI ≥30, chronic kidney disease, chronic pain, chronic obstructive pulmonary disease (COPD), congestive heart failure, arrhythmia/atrial fibrillation, and pulmonary heart disease. We originally planned to recruit participants based on self-reports of the first 5 conditions (4 of them related to metabolic syndrome). Before recruitment began, we changed our criterion to 3 or more of any of the 11 conditions listed above. We did so both to increase the pool of eligible patients and to provide a more expansive test of the effectiveness of ET in addressing a wide array of comorbid conditions prevalent among older adults.

Prior to recruitment, we also decided to use documented diagnoses from electronic health records (EHRs) as inclusion criteria, rather than relying on participant self-report (including possible self-diagnosis). This not only provided a more standardized assessment of chronic conditions but also allowed clinics to identify eligible participants and send them a recruitment letter describing the study.

Further eligibility criteria were as follows: (4) no current psychotic disorder that would prevent participation; (5) no acute medical problem requiring immediate hospitalization; (6) no visual or motor impairment preventing the use of a computer; (7) ability to read and sign the consent form in English; (8) agreement to share health-related study data (eg, lab scores and health care utilization); (9) permission to share information with the patient’s primary care physician; and (10) no moderate or advanced dementia.

### Interventions

Patients in both conditions continued with their UC provided by primary care and internal medicine clinics in the University of Wisconsin–Madison system and received their assigned intervention.

#### Control Condition

In addition to their UC, patients in the control condition received internet service and a laptop computer for 12 months. Shortcuts to 4 general health information websites, vetted for quality by our research team, were placed on the computer desktop for easy access: the Cleveland Clinic [45], National Institute on Aging [46], the American Academy of Family Physicians [47], and Mayo Clinic [48]. We expected this UC+internet intervention to be relatively ineffective because information alone is unlikely to have much impact on health behaviors [39,49-51]. Instead, access to the device, the internet, and the sites functioned as a form of attention control versus pure control comparison and as a way to isolate the specific effects of access to ET. In an attention control group, participants receive the same amount of interaction and “attention” from the research team as the intervention group, but that interaction does not contain the hypothesized therapeutic content [52]. In our case, control participants could go online and interact with others but did not do so in the context of ET.

#### Experimental Condition

Patients in the experimental condition received UC plus ET access and a laptop computer, along with internet for 12 months. This UC+ET group did not receive the health information websites placed on computers for control patients, although they could have sought them out independently.

#### ET System Overview

As described previously [26], ET provides tools, motivation, and social support to help patients manage their chronic conditions, communicate with peers and research staff, and improve communication with clinicians. The system was developed specifically for older adults with extensive input from advisory and focus groups of older adults. It featured large fonts, few options, and uncluttered screens for easy comprehension, navigation, and usability. Areas of the site were Community (with chatroom-like discussion groups, email-like private messaging, and a bulletin board of local events); Well-Being (offering relaxation and meditation videos and journaling with positive psychology prompts); and My Health (a collection of health information resources). The content was continuously refreshed. While the design and navigation were based on the original ET and principles established in our earlier testing [27,53], we enhanced the system for this study as described below. A sample of the home screen is shown in Figure S1 in Multimedia Appendix 2.

#### ET Enhancements

We made 3 major enhancements to the original ET. First, we expanded a basic health-tracking feature in the original ET to create a weekly survey with feedback. Patients were prompted to complete weekly check-ins regarding 10 general health indicators: sleep, nutrition, physical activity, cognition, balance, falls, mood, pain management, medication adherence, and quality of social interactions. As soon as the survey was completed, ET feedback commended any improvements. If survey results showed mild reductions in any of the health indicators, ET feedback directed the patient to relevant articles in the ET My Health library. If the algorithm detected a sudden or steep negative change in any of the indicators or a problem that was not improving over several weeks, ET recommended that the patient contact their clinic. ET also generated a graph charting the patient’s responses for each indicator over the last 3 months to facilitate self-monitoring (Figure S2 in Multimedia Appendix 2).

Second, we used the survey-generated graph as the basis for a clinician report that was shared with the patient’s primary care clinic. We used a similar report in a lung cancer trial comparing a CHESS system alone to a CHESS system combined with a clinician report [54] and found that the addition improved symptom distress by more than 100% (26.2% improvement with CHESS alone vs 53% with CHESS+clinician report; n=71 vs 68, respectively; *P*<.001). The clinician report was designed to offer specific benefits in health care delivery for patients with MCCs. While MCCs can lead to rapid declines in health [55], care for such patients usually consists of periodic onsite contact with primary care clinicians, who may be unaware of such changes or cannot respond as promptly as may be warranted. The report, sent the week before a patient’s scheduled visit, shared timely information on health indicators and helped both patients and clinicians make the most of these visits. As a single-page graphic summary of health-tracking data, the report could be viewed and understood at a glance, avoiding a time burden for clinicians while allowing them to provide responsive and appropriate treatment. A hard copy of the report was also mailed directly to the participant to take to the appointment.

When the COVID-19 lockdown began and clinics transitioned to telehealth and fewer in-person visits, we altered our strategy for the clinician report. Every 2 months, the study project manager prepared a clinician report summary providing an overview for all participants at each clinic. The goal was to help clinicians identify patients who were reporting issues such as missed medications or mood changes between appointments, particularly as patients continued to isolate due to COVID-19. This summary was emailed to clinic managers to share with individual clinicians (Figure S3 in Multimedia Appendix 2).

Third, because our original trial of ET identified the greatest improvements in mental QOL, social support, and depression, we tried to strengthen these effects still further by adding an interactive Fun & Games area (light-hearted polls, quizzes, videos, and games) and a weekly Lifestyle article (topics included travel, mind and body, etc). Both features were designed to increase participants’ enjoyment of the site and engagement with others via asynchronous comment threads.

### Measures

Participants were assessed for all study variables at baseline, 6 months, and 12 months, as described below. To assess system use, time-stamped usage data from ET participants were continuously captured in our database, including specific services used; date and time the system was accessed; and text entered into discussions, comment threads, and message features.

#### Primary Outcomes

Mental and physical QOL were assessed using the 8-item PROMIS (Patient-Reported Outcomes Measurement Information System) Global Health measure [56,57], with 4 items for mental QOL (eg, “How would you rate your mental health, including your mood and your ability to think?”) and 4 for physical QOL (eg, “To what extent are you able to carry out your everyday physical activities such as walking, climbing stairs, carrying groceries, or moving a chair?”). Scoring of individual items varies and is calculated by the scale’s developers; the total possible ranges are 21.2-67.6 for mental QOL and 16.2-67.7 for physical QOL, with higher values indicating better QOL [58]. For consistency with other primary outcome measures, the timeframe was modified to the past 2 weeks.

Psychological well-being was assessed with the 8-item Psychological Flourishing Scale (eg, “My social relationships are supportive” and “I lead a meaningful life”) [42]. Each item is scored on a 5-point scale, for a total possible range of 8-40, with higher scores indicating greater flourishing.

Loneliness was measured with 8 items from the UCLA Loneliness Scale that showed the highest factor loadings among elderly adults in prior work (eg, “How often do you feel part of a group of friends?”) [59]. Each item is scored on a 5-point scale, for a total possible range of 8-40, with higher scores indicating increasing loneliness.

#### Mediators

We measured 5 possible mediators. Health coping strategies were assessed with 10 items from the Ways of Coping Scale (eg, “Made a plan of action and followed it” and “Accepted the situation”) [60]. Each item is assessed on a 5-point scale, for a possible range of 10-50, with higher scores indicating better coping.

Motivations were assessed with four 5-point items from the Treatment Self-Regulation Questionnaire [61]; 2 items assessed autonomous motivation (eg, “I try to manage my health conditions because I want to take responsibility for my own health”) and 2 assessed external regulation (eg, “I try to manage my health conditions because I feel pressure from others to do so”). The total possible range for each motivation subscale is 2-10, with higher scores indicating greater autonomous and external motivation.

Relatedness was assessed with the 6-item McTavish Bonding Scale [62] plus 3 items from the short form of the PROMIS emotional support scale [63]. For all items, patients indicated on a 5-point scale the frequency of the type of support (eg, “Someone you can count on to listen when you need to talk” and “Someone to love and make you feel wanted”). The total possible range is 9-45, with higher scores reflecting more support.

Anxiety and depressive symptoms were assessed with the 7-item Generalized Anxiety Disorder scale [64] and the 8-item version of the Patient Health Questionnaire Depression scale [65], respectively. The response option in both 4-point scales was the frequency of a symptom (eg, “Trouble relaxing” and “Poor appetite or overeating”) in the past 2 weeks. Higher scores indicate more anxiety or depression symptoms.

#### Moderators

We investigated whether the effects of the study arm on change from baseline to endpoint in primary outcomes were moderated by sex, health care use, and number of chronic conditions. Participants indicated their sex (male or female) at baseline. On all assessments, they reported scheduled visits in the preceding 3 months to primary care, specialists, physical and occupational therapists, chiropractors, and counseling. For the number of chronic conditions, we used EHR data obtained during enrollment for each participant, and the range was 3-11.

#### Potential Covariates

Potential covariates included comfort or familiarity with technology, physical challenges using technology, and life stressors. Patients rated on a 6-point scale their comfort with 6 communication technologies: computer, smartphone, tablet, smart speaker, email, and Facebook. The total possible range is 0-30, with higher scores indicating greater comfort. For physical challenges, participants reported vision, hearing, hand pain or tremors, memory, “other,” or no limitations for both a computer/tablet and a smartphone, checking all that applied. To gauge life stressors, they reported on 14 possible stressors from the Social Readjustment Rating Scale (eg, “Death of a very close friend or family member” and “Change in financial status”) [66], checking all that applied (possible range is 0-14). Participants also reported sociodemographic variables of race and ethnicity, education, income, health insurance, whether they had a significant other, housing type, and whether they lived alone or with others. We planned to control for these if they varied by study arm.

### Sample Size Determination and Power

We focused on the effect of Cohen d=0.50 on the primary outcome of patients’ perceptions of their QOL, given recommendations that this is the minimally important difference for QOL measures in clinical trials [67]. For our other primary outcomes (psychological well-being and loneliness), effect sizes tend to be smaller. For example, a prior web-based intervention for rural women with chronic diseases showed an effect of Cohen d=0.29 on loneliness among those who scored above the median on baseline levels of loneliness, depression, and stress [68]. Given that our intervention was substantially longer (12 months vs 22 weeks) and had more components specifically designed to address social connectedness, we expected somewhat larger effects but did not expect to reach Cohen d=0.50. Balancing the need to be adequately powered with the need to focus on meaningful impacts, the study was powered to detect a main effect of Cohen d=0.35 for our primary outcomes. Adequate power to detect a between-subjects effect of Cohen d=0.35 (1−β=0.80; α=.05) with repeated measures on the outcome required a final sample of 262 patients (130 per arm). On the basis of our prior trial of ET, we assumed 20.5% attrition and thus arrived at a recruitment goal of 330 patients (165 per arm).

### Recruitment

The UW–Madison Clinical Research Data Service examined UW Health Clinic EHRs to identify patients who met the eligibility criteria, including chronic conditions. Potential participants then received an opt-in letter from the university’s Office of Clinical Trials. The letter described the study and included a postage-paid return invitation for further contact from the study team.

After a patient returned the invitation, study staff called and provided a detailed trial overview, including benefits and potential risks of participation. If interested, patients were screened on all eligibility criteria. Those who verbally confirmed they wanted to participate and met the criteria were mailed the baseline survey and received a home visit from a member of the research team, at which time written consent was obtained, baseline data were collected, and randomization was determined.

### Randomization

The project manager used a computer-generated allocation sequence to randomize patients in a 1:1 ratio to the experimental (UC+ET) or control (UC+internet) group, stratified by sex, clinic site, and number of chronic conditions (3-5 vs. ≥6). The block size was 10. When baseline assessment and consent were complete, the research staff person opened the numbered, sealed, opaque envelope revealing group assignment, and then conducted equipment setup and training for either the intervention (including all the services and how to use them) or control device. Once the assignment was made, participants could not be blind to their condition, given that those in the experimental arm were asked to use ET, while those in the control arm were not. The training researcher also could not be blind to the condition after the assignment was revealed.

### Statistical Methods

From the list of our prespecified potential covariates, we selected covariates to include in analyses by examining whether they were moderately correlated (0.30 or higher) with at least one of the primary outcomes [69]. Only life stressors met the correlation benchmark (mental QOL: r=–0.35; physical QOL: r=–0.34).

In addition, given that participants varied in the extent of overlap between the COVID-19 pandemic and the 12-month intervention period (range=0.02 to 6.33 months), we included this variable (defined as participant’s number of months during the pandemic prior to each survey timepoint) in our analyses at each timepoint as a covariate. Of note, pandemic months significantly influenced mental QOL (*P*=.03) but none of the other primary outcomes (all *P*>.31). Ultimately, life stressors and pandemic months were the only covariates included in the models.

Normality, linearity, and homoscedasticity/homogeneity of variance for outcome data were assessed using descriptive statistics and graphical representations. Outcomes were analyzed with mixed-effects models, using (g)lmer() from the lme4 package, implemented in the R statistical software environment. These models account for correlated measurements within participants, use all available data (allowing for intention-to-treat rather than only complete-case analysis), and provide unbiased estimates when data are missing at random [70]. Each model included a random effect for participant, as well as fixed effects for survey timepoint, study arm, and arm-by-timepoint interaction. The survey timepoint was entered as a continuous variable with 3 timepoints (baseline, 6 months, and 12 months).

### Ethical Considerations

This study received ethical approval from the University of Wisconsin Health Sciences Institutional Review Board (reference number: 2017-0849) and has been registered at ClinicalTrials.gov (NCT03387735).

## Results

### Participants

**Figure 2:**
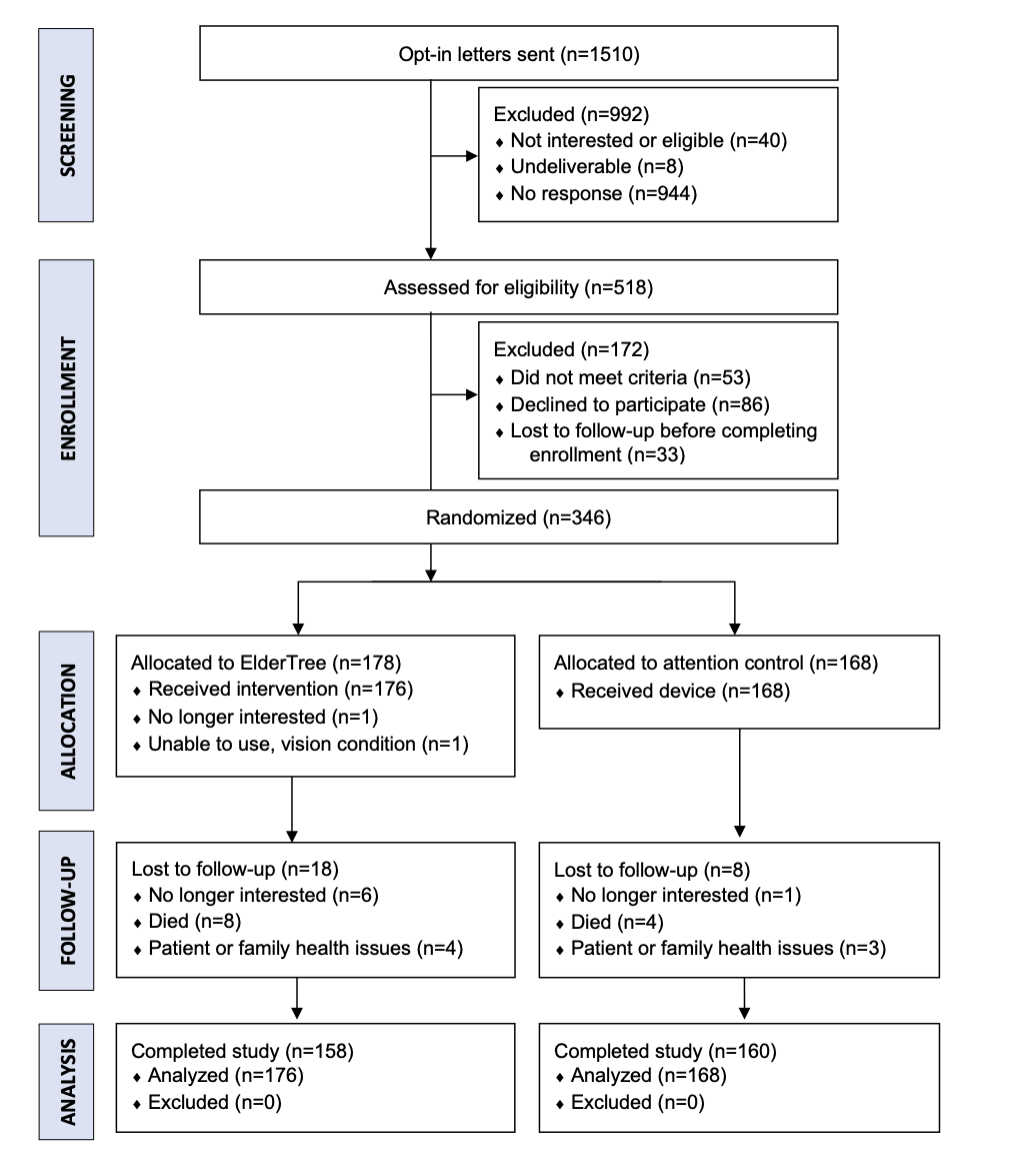
CONSORT (Consolidated Standards of Reporting Trials) flow diagram of participants through the study. Data were analyzed with mixed-effects models, which use all available data rather than only complete cases.

A total of 346 participants were randomized, and 344 received either the assigned intervention or control device and were included in the analyses (Figure 2). Of the 344 receiving an intervention, 321 (93.3%) completed the 6-month survey and 309 (89.8%) completed the 12-month survey. As data were analyzed with mixed-effects models, which use all available data rather than only complete cases, 318 (92.4%) were included in the analyses. Recruitment began in February 2018 and ended in December 2019. The intervention period concluded in December 2020 (Figure 2). The CONSORT (Consolidated Standards of Reporting Trials) checklist is presented in Multimedia Appendix 3.

Participant characteristics at baseline are presented in Table 1. Most participants identified as white (317/344, 92.2%) and female (209/344, 60.8%), and the average age was 74 years 9 months. The average number of chronic conditions was 5.28. For a detailed breakdown of chronic conditions by group, see Multimedia Appendix 4.

**Table 1:** Participant characteristics by study arm at baseline.

Characteristic			UC^a^+internet (N=168)		UC+ElderTree (N=176)
Female, n (%)			103 (61.3)		106 (60.2)
**Number of chronic conditions at enrollment (from EHRs^b^), n (%)**					
	3-5 chronic conditions	104 (61.9)		108 (61.4)	
	6-11 chronic conditions	64 (38.1)		68 (38.6)	
**Race, n (%)**					
	American Indian, White	1 (0.6)		2 (1.1)	
	Black	14 (8.3)		8 (4.6)	
	Jewish	0 (0.0)		1 (0.6)	
	Unspecified mixed race	0 (0.0)		1 (0.6)	
	White	153 (91.1)		164 (93.2)	
Ethnicity Hispanic or Latino, n (%)			1 (0.6)		0 (0.0)
**Highest level of completed education, n (%)**					
	Middle school	2 (1.2)		1 (0.6)	
	High school	40 (23.8)		30 (17.1)	
	Vocational or technical school	19 (11.3)		26 (14.8)	
	Some college	38 (22.6)		49 (27.8)	
	College graduate	30 (17.9)		35 (19.9)	
	Postgraduate or professional	39 (23.2)		35 (19.9)	
**Annual income, n (%)**					
	Less than US $12,000	5 (3.0)		7 (4.0)	
	US $12,000-24,999	34 (20.2)		32 (18.2)	
	US $25,000-49,999	44 (26.2)		41 (23.3)	
	US $50,000-74,999	30 (17.9)		34 (19.3)	
	US $75,000 or above	41 (24.4)		43 (24.4)	
	Did not report	14 (8.3)		19 (10.8)	
Had significant other, n (%)			105 (62.5)		110 (62.5)
**Housing, n (%)**					
	Nursing home	1 (0.6)		0 (0.0)	
	Own	114 (67.9)		134 (76.1)	
	Rent	33 (19.6)		27 (15.3)	
	Assisted living facility	3 (1.8)		1 (0.6)	
	With family or friends	11 (6.6)		11 (6.3)	
Live alone, n (%)			118 (70.2)		124 (70.5)
Age (years), mean (SD)			74.54 (6.45)		74.88 (6.11)
Comfort using technology (range 0-5)^c^, mean (SD)			2.81 (1.30)		2.79 (1.34)
Physical issues with technology (range 0-10)^d^, mean (SD)			0.46 (1.19)		0.38 (0.87)
Life stressors (range 0-14)^e^, mean (SD)			1.11 (1.46)		1.01 (1.36)

^a^UC: usual care.

^b^EHRs: electronic health records.

^c^Higher values indicate greater comfort.

^d^Higher values indicate more physical issues.

^e^Higher values indicate more stressors.

### ET Use

We defined “use” as accessing any service or feature beyond the home screen at least once during a given time period. Of the 176 participants randomized to UC+ET, 166 (94.3%) had used ET in their first month, 164 (93.2%) used it in months 2-6, 137 (77.8%) used it in months 7-11, and 134 (76.1%) used it in the final month of the study.

Number of days of use (ie, accessing any ET service in a 24-hour period) also indicated sustained use. In the first 6 months (180 days), participants accessed ET for a mean of 48.95 days (SD 35.16 days; 27.2% of days; range 0-179 days). During months 7-12, participants used the intervention for a mean of 31.40 days (SD 32.07 days; 17.44% of days; range 0-167 days). These means include participants with no days of use (scored 0).

Participants made the most use of ET services that facilitated social interaction. Of the 6 main areas on the site, Community was the most active. During their year on the intervention, 172 of the 176 UC+ET participants used this area a mean of 42.78 days (SD 59.66 days; range 1-305 days). Discussion Groups, an activity within the Community area, was the most heavily used single service on the system. During their intervention year, a total of 162 participants used it for a mean of 34.82 days (SD 57.48 days; range 1-295 days). Of the other main areas, Fun & Games was the second most visited (n=163 participants; mean 32.74 days, SD 57.50 days; range 1-311 days), followed by Lifestyle (n=163; mean 15.34 days, SD 23.81 days; range 1-214 days), both of which allowed for asynchronous interaction via comments. The nonsocial areas were used somewhat less often: My Health informational resources were used by 169 participants for a mean of 24.89 days (SD 21.66 days; range 1-135 days), and Well-Being was used by 158 participants for a mean of 15.32 days (SD 35.14 days; range 1-290 days).

Regarding the weekly health-tracking feature, 162 of the 176 UC+ET participants (92.0%) used the tracker during their first 6 months in the study, and 134 (76.1%) were still using it in the 12th month. Prior to the 6-month survey, participants used the tracker for a mean of 20.16 weeks (SD 7.11 weeks; range 0-30 weeks). Between the 6- and 12-month surveys, participants used it for a mean of 16.99 weeks (SD 8.21 weeks; range 0-26 weeks). These means include participants with no weekly tracker use (scored 0).

### Use of the Clinician Report and Summaries

We had planned to conduct study-end quantitative assessments and qualitative interviews with clinicians about their uses and perceptions of the clinician report. Because of the pandemic, clinicians were unavailable to provide feedback on either the prepandemic individual reports or the clinic summaries we pivoted to once the pandemic started. We had anecdotal feedback from staff members that the clinic summaries were helpful in highlighting patients who might need outreach, but we had no way of assessing the extent to which clinicians used the reports or summaries and which aspects they found helpful or cumbersome. We did not measure patients’ uses and perceptions of the clinician reports, but some participants volunteered instances in which the clinician report sparked a conversation with their doctor (eg, about sleep).

### Effects of the Study Arm on Changes Over Time in Primary Outcomes

Primary outcomes were physical and mental QOL, psychological well-being, and loneliness. We conceptualized these as distinct though related variables. In fact, the 3 socioemotional outcomes (mental QOL, psychological well-being, and loneliness) were more strongly correlated than we had anticipated (Table 2).

**Table 2:** Correlations between primary outcomes.

Variable	Data			Correlation			
	Number	Mean (SD)	Mental quality of life		Physical quality of life	Psychological well-being	Loneliness
Mental quality of life	344	46.25 (7.28)	—^a^		0.50^b^	0.61^b^	–0.56^b^
Physical quality of life	344	42.39 (6.23)	0.50^b^		—	0.37^b^	–0.17^b^
Psychological well-being	344	31.33 (5.23)	0.61^b^		0.37^b^	—	–0.68^b^
Loneliness	344	16.59 (5.93)	–0.56^b^		–0.17^b^	–0.68^b^	—

^a^Not applicable.

^b^*P*<.05.

**Figure 3:**
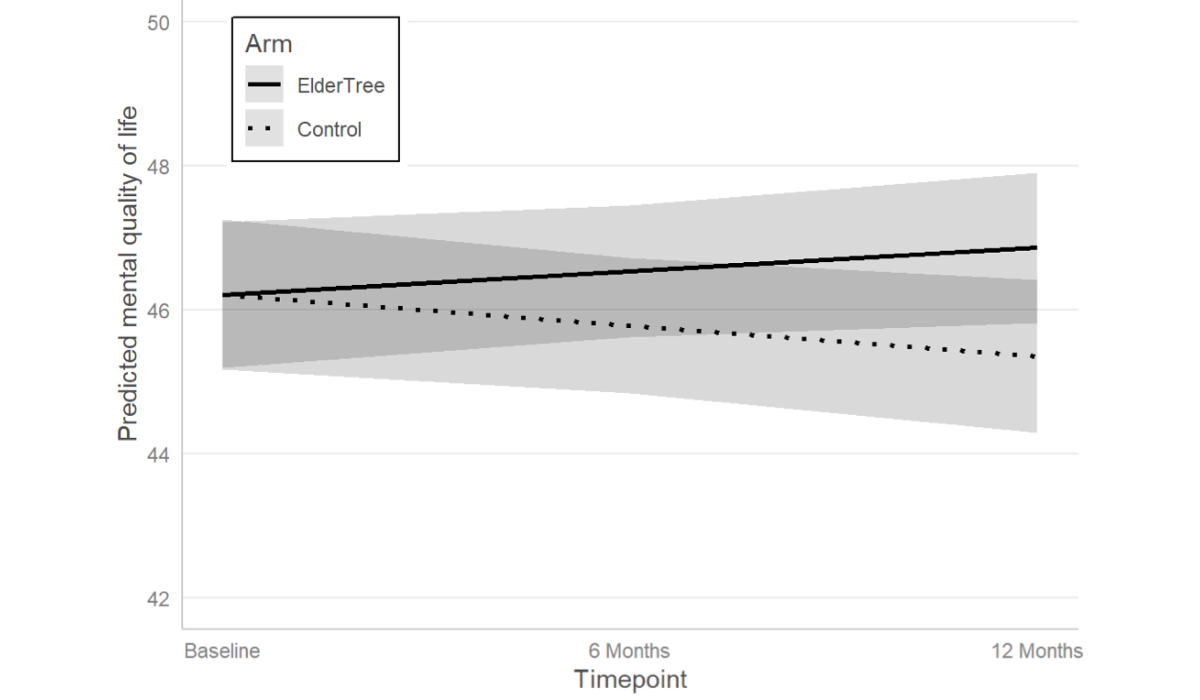
Predicted mean values of mental quality of life over time. Possible range is 21.2-67.6, with higher values indicating better mental quality of life. Shaded areas represent 95% CIs.

Controlling for life stressors and pandemic months, we found a significant difference between the ET and control arms in the extent of change in mental QOL over time from baseline to 12 months (arm × timepoint interaction: b=0.76, 95% CI 0.14-1.37; *P*=.02). Specifically, as shown in Figure 3, the ET arm showed an increase in mental QOL and the control arm showed a decrease, for a total difference of 1.5 T-score points (PROMIS measures are standardized so that scores have a mean of 50 and SD of 10. Thus, the T-score difference can be interpreted as Cohen d divided by 10, that is, 1.5 points can be read as Cohen d=0.15). Although this difference in means between the 2 groups at 12 months was statistically significant (*P*=.04), PROMIS scoring guidelines [71,72] state that a difference is considered meaningful at 3 or more T-score points when comparing groups.

We did not find significant differences between groups in changes over time for physical QOL (b=0.10, 95% CI –0.44 to 0.64; *P*=.71), psychological well-being (b=0.23, 95% CI –0.26 to 0.72; *P*=.36), or loneliness (b=–0.25, 95% CI –0.73 to 0.22; *P*=.29).

### ET System Use as a Predictor of Primary Outcomes

As the overall goal of the study was to assess the effects of ET, we examined whether the amount of use of the ET system would predict changes in our primary outcomes, as specified in our protocol [26]. For these analyses, the amount of ET use was measured as days of any use. If a participant in the intervention arm did not use the system after training, they were assigned a value of 0 days. Both arms (control and intervention) were included in these analyses, with all control participants assigned values of 0 days.

With regard to mental QOL, controlling for baseline scores, days of ET use significantly predicted improvement over the 12 months of the intervention (b=0.02, 95% CI 0.00-0.03; *P*=.046), and the magnitude of this effect did not change over time (b=0.00, 95% CI –0.02 to 0.02; *P*=.72). The model predicted that those using ET every day within a 6-month time span (180 days) would show a 3.23-point increase in mental QOL compared to those not using the system. A clinically meaningful difference of 3 points would thus be seen at 167 days within a 180-day span or 92.7% of days.

Days of ET use did not statistically predict a difference in the other primary outcomes: physical QOL (b=–0.00, 95% CI –0.02 to 0.01; *P*=.90), psychological well-being (b=0.01, 95% CI –0.01 to 0.02; *P*=.26), or loneliness (b=–0.01, 95% CI –0.02 to 0.00; *P*=.20).

### Mediation of the Effects of the Study Arm

As proposed, we investigated whether the effects of the study arm on changes in primary outcomes over time would be mediated by 6-month changes in health coping strategies, health-related motivation, feelings of relatedness, depression symptoms, and anxiety symptoms. Controlling for baseline, the study arm did not significantly predict coping (b=–0.69, 95% CI –2.14 to 0.75; *P*=.34), motivation (b=0.04, 95% CI –0.56 to 0.64; *P*=.88), depression (b=0.05, 95% CI –0.53 to 0.64; *P*=.86), or anxiety (b=0.12, 95% CI –0.48 to 0.71; *P*=.70) at 6 months (simple test of path α). However, the study arm did significantly predict feelings of relatedness (b=1.35, 95% CI 0.13-2.57; *P*=.03) at 6 months. Given this, we tested a simple mediation model of the effect of the study arm on the change from baseline to 12 months in our primary outcome mental QOL, mediated by the change from baseline to 6 months in relatedness. We did not test mediation for our other 3 primary outcomes because the arm-by-time analyses were nonsignificant.

**Figure 4:**
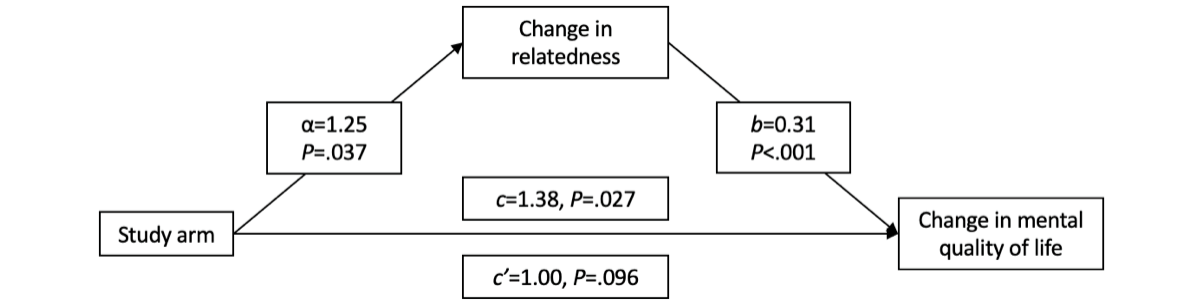
Diagram of the study arm mediational model.

The mediation analysis showed that the study arm was significantly associated with changes in mental QOL from baseline to 12 months (path *c*=1.38; *P*=.03), as was demonstrated by our main primary outcome analyses. The inclusion of relatedness in the model resulted in significant associations between the study arm and relatedness (path α=1.25; *P*=.04) and between relatedness and mental QOL (path *b*=0.31; *P*<.001). The association between the study arm and changes in mental QOL (direct effect) was not significant (path *c*′=1.00; *P*=.10), indicating complete mediation. See Figure 4 for a diagram of this mediation model.

### Moderation of the Effects of the Study Arm

We examined whether the effects of the study arm on changes over time in the primary outcomes were moderated by sex, amount of scheduled health care use, and number of chronic conditions.

**Figure 5:**
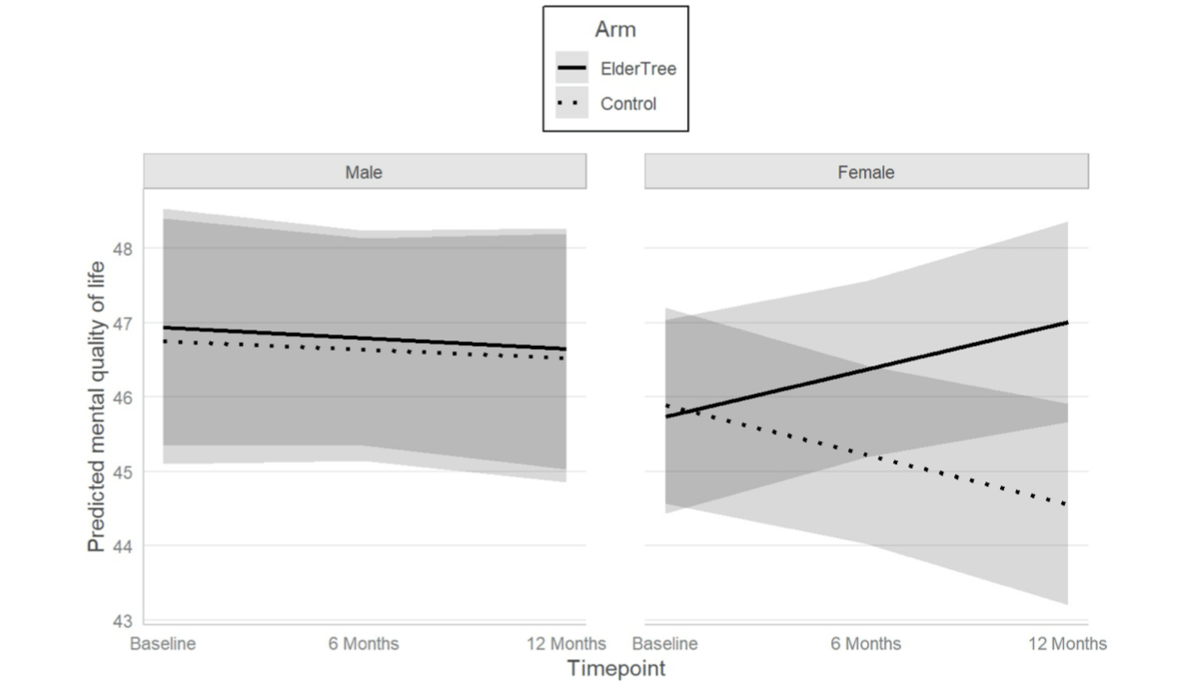
Predicted mean values of mental quality of life by sex over time. Possible range is 21.2-67.6, with higher values indicating better mental quality of life. Shaded areas represent 95% CIs.

Sex moderated the effect of the study arm over time on mental QOL (sex × arm × time interaction: b=1.33, 95% CI 0.09-2.58; *P*=.04). To understand this 3-way interaction, we looked at the simple effects of time for the 4 combinations of sex and arm. For female participants, the ET arm showed an increase in mental QOL over time, while the control arm showed a decrease. By 12 months, the difference in means was statistically significant (mean difference=2.60; *P*=.002) for females, although it did not exceed the benchmark of 3.0 considered meaningful. For male participants, no difference in means was seen between groups over time (12-month mean difference=–0.06; *P*=.95). Data are presented in Figure 5.

**Figure 6:**
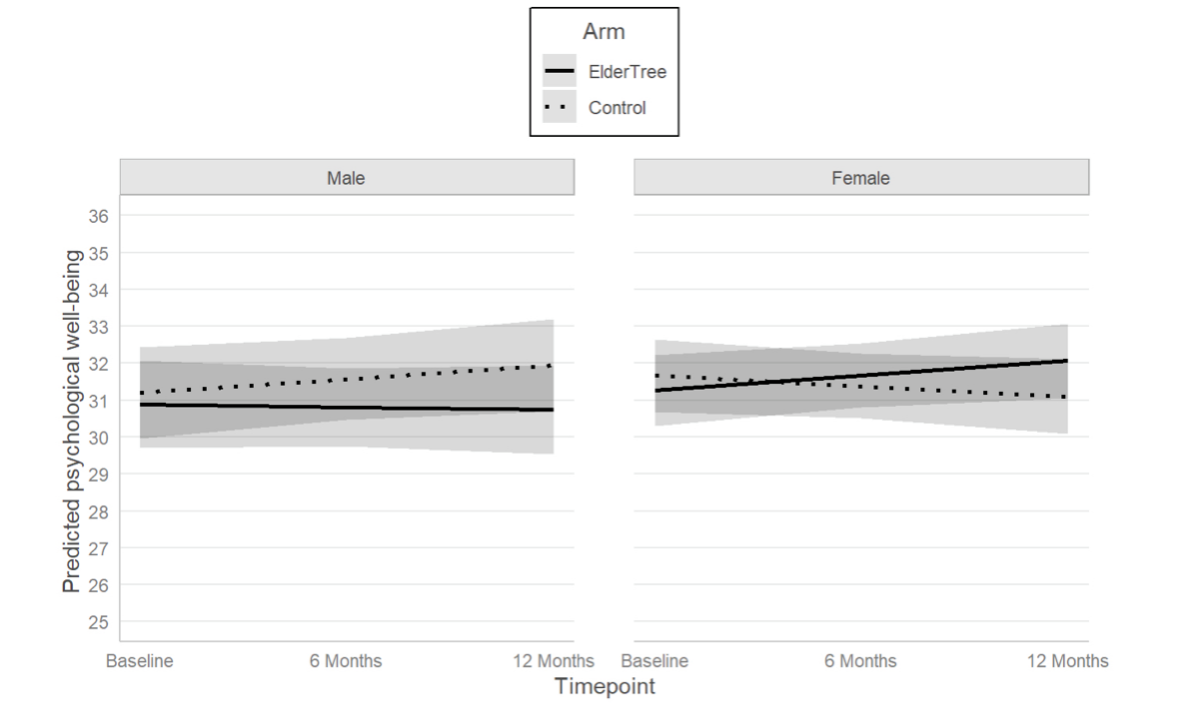
Predicted mean values of psychological well-being by sex over time. The possible range is 8-40, with higher values indicating greater psychological well-being. Shaded areas represent 95% CIs.

Sex also moderated the effect of the study arm over time for psychological well-being (sex × arm × time interaction: b=1.13, 95% CI 0.13-2.12; *P*=.03). To understand this 3-way interaction, we looked at the simple effects of time for the 4 combinations of sex and arm. For female participants, the ET arm showed an increase in well-being over time and the control arm showed a decrease. By 12 months, the difference in means was statistically significant (mean difference=1.38; *P*=.04; Cohen d=0.28). For male participants, the ET arm remained steady while the control group showed an increase in psychological well-being over time. However, at 12 months, the difference in means was not statistically significant (12-month mean difference=0.88; *P*=.28; Cohen d=0.18). Data are presented in Figure 6.

No other moderation of the effect of the study arm over time was found. Inferential statistics for all moderation analyses are presented in Table 3.

**Table 3:** Inferential statistics for the moderators of the study arm regarding primary outcomes.

Moderator and outcome		Inferential statistics for moderator × arm × timepoint		
		b	95% CI	*P* value
**Sex**				
	Mental quality of life	1.33	0.09 to 2.58	.04
	Physical quality of life	0.08	–1.02 to 1.18	.88
	Psychological well-being	1.13	0.13 to 2.12	.03
	Loneliness	0.51	–0.45 to 1.48	.30
**Number of scheduled health care visits**				
	Mental quality of life	–0.06	–0.17 to 0.04	.26
	Physical quality of life	0.00	–0.09 to 0.10	.92
	Psychological well-being	–0.05	–0.13 to 0.04	.28
	Loneliness	–0.00	–0.09 to 0.08	.91
**Number of chronic conditions from EHRs^a^**				
	Mental quality of life	–0.02	–0.49 to 0.45	.94
	Physical quality of life	–0.01	–0.42 to 0.41	.97
	Psychological well-being	–0.02	–0.40 to 0.35	.91
	Loneliness	–0.01	–0.38 to 0.35	.94

^a^EHRs: electronic health records.

### Post Hoc Probing of Sex Moderation

To probe the finding that the effects of the study arm on mental QOL and psychological well-being were stronger for women than for men, we began by examining whether there were sex differences in variables that might explain this pattern: other demographic or background variables that we had considered as covariates, baseline levels of relatedness and primary outcomes, days of ET use, and (more specifically) days of ET discussion group use. There were only 2 significant differences. For education (4=some college, 5=college graduate, 6=postgraduate or professional degree), men (mean 5.37, SD 1.36) had higher levels than women (mean 4.85, SD 1.46; *P*<.001). For the likelihood of having a partner, men (n=112, 83%) had a higher likelihood than women (n=103, 49%; *P*<.001). However, adding education and the presence of a partner as covariates to the moderation analyses for mental QOL and psychological well-being did not alter the significance of the study arm × time × sex interactions or the magnitude or direction of the coefficients for the study arm (b=1.35, 95% CI 0.10-2.60; *P*=.03 for mental QOL; b=1.14, 95% CI 0.15-2.14; *P*=.025 for psychological well-being). See Table 3 for values without education and partner. Further exploratory analyses examining possible moderated mediation are reported in Multimedia Appendix 1.

### Post Hoc Moderation Analysis: Effect of COVID-19 on the Study Arm for Primary Outcomes

Because our outcomes seemed likely to be impacted by the extended isolation of lockdowns, which began in March 2020, and because our older health-compromised population was among the most vulnerable to the illness [73,74], we conducted post hoc analyses. Specifically, to understand how the pandemic may have affected primary outcomes, we tested pandemic months as a moderator.

The pandemic overlapped with some portion of the 12-month intervention period for 158 (45.9%) of the 344 participants who received an intervention. A total of 77 (43.8%) ET and 81 (48.2%) control participants completed the 12-month survey during the pandemic, and of these, 3 (1.7%) ET and 6 (3.6%) control participants also completed the 6-month survey during this period. The 81 control participants experienced a mean overlap of 3.11 months (SD 2.03 months; range 0.02-6.33 months), while the mean overlap for the 77 ET participants was 3.02 months (SD 1.94 months; range 0.20-6.10 months). Given this, we examined months of overlap with the pandemic as a possible unanticipated moderator (rather than covariate) of the effects of the study arm.

The pandemic did not appear to impact the effects of the study arm on the primary outcomes. Controlling for baseline, we found that months during COVID-19 did not moderate the effect of the study arm for mental QOL (b=–0.98, 95% CI –4.03 to 2.08; *P*=.53), physical QOL (b=–0.13, 95% CI –2.91 to 2.64; *P*=.93), psychological well-being (b=–1.73, 95% CI –4.16 to 0.71; *P*=.16), or loneliness (b=1.15, 95% CI –1.27 to 3.57; *P*=.35).

### Secondary and Exploratory Outcomes

Results for secondary and exploratory outcomes are presented and discussed in Multimedia Appendix 1. To summarize, we found a significant effect of the study arm on changes over time in the level of pain, such that the ET arm showed a slight increase in pain over time, while the control arm showed a slight decrease, resulting in a significant difference between study arms at 12 months. We did not find significant effects of ET versus control on changes over time in falls, symptom distress, medication adherence, use of crisis health care, pain medication issues, alcohol use problems, or diet. We were unable to test cigarette use due to a lack of participants who smoked at any point during the study.

## Discussion

### Summary and Interpretation

Given the need for broadly applicable behavioral interventions that could be implemented in primary care to help manage patients’ varied combinations of chronic conditions [20,21], this study was designed to test the effectiveness of an online intervention, ET. Given that prior interventions for multimorbidity have been critiqued for low-quality assessments [25], this study was designed to be a high-quality clinical trial featuring a large sample of older adults with 3 or more of 11 chronic conditions randomized to the ET intervention or an attention control group for a full year. The goals of the intervention were to help older adults manage their chronic conditions (in part by providing their primary care team with insights about their ongoing health status) and to connect them with peers for social support. A previous iteration of ET yielded positive effects on socioemotional outcomes of mental QOL, social support, and depression among those with high levels of primary care use [27]. The current iteration leaned into this finding, with system enhancements designed to connect participants with their primary care team and to strengthen peer interactions and pleasurable aspects of the site.

Consistent with the findings for the earlier version of ET, the current ET version showed signs of improving socioemotional outcomes. There was a significant effect of ET (vs control) on improvements over the 12-month intervention period regarding the primary outcome of mental QOL, as well as a significant study arm × sex interaction, indicating stronger effects for women than men. There was a significant moderated effect of ET on improvements over the 12-month intervention period in the primary outcome of psychological well-being, again indicating stronger effects for women than men. There was a significant effect on improvements over 6 months in relatedness, which was our hypothesized mediator variable that assessed feelings of social support, love, and connectedness. 

As such, even though we did not find the predicted effects on the primary outcome of loneliness, ET relative to an attention control group showed socioemotional benefits for older adults with MCCs. We acknowledge that the 3 socioemotional primary outcome variables (mental QOL, psychological well-being, and loneliness) were more strongly correlated than we initially anticipated. Given that we had preregistered the analyses in our protocol paper and given that we continue to believe these are important and conceptually distinct variables, we have treated them as separate variables. However, it is important to note that doing so inflates the risk of type 1 errors across the set of outcomes, and therefore, the identified effects should be considered preliminary until replicated.

It is also important to acknowledge that the difference between study arms for mental QOL at 12 months was smaller than the benchmark for a clinically meaningful effect (benchmark = T-score difference of 3.0), both in the overall sample (difference=1.5) and for women (difference=2.6). Our analyses examining days of ET use predicted that the benchmark difference between study arms would occur with at least 167 days of app use over 6 months (or at least 93% of days), suggesting that while clinically meaningful effects for mental QOL may be possible, substantial engagement with ET would be needed. Possible strategies to foster sustained high levels of engagement are being examined in our subsequent ongoing RCT of ET. Our current iteration of ET is now being tested on smart displays as well as laptops, the content library is much larger, and we now have weekly video-chat “meet-ups.” These meet-ups not only allow participants to interact with each other and build social connections but also provide opportunities for a trained moderator to highlight key aspects of ET and their uses.

Although participants’ engagement with ET was markedly lower than our analyses suggest would be needed for clinically meaningful effects on mental QOL, it is noteworthy that the percentage of participants who were still using ET at the end of the year-long intervention (76.14%) was far higher than is typical for mental health apps. A 2019 review of 93 apps targeting emotional well-being, anxiety, or depression found median 30-day retention rates of 3.3%, with somewhat higher rates for 2 apps offering peer support (8.9%) [75]. A 2022 review of 56 mental health apps found that retention 7 days after download varied from 5.5% to 19% [76]. Although ET use was undoubtedly higher because participants were aware of being in a study, they were not required to use the app. The fact that they showed the most use of the tools for social engagement suggests that these forms of contact continued to have meaning and value for them across the year of the study.

In contrast to the effects on a subset of socioemotional outcomes, we found no significant effects of ET on any of our physical health outcomes. Due to insufficient EHR data, as described in Multimedia Appendix 1, we were unable to conduct planned analyses for participants’ lab scores. However, there were no significant effects of ET on participants’ physical QOL or the secondary outcome of “symptom distress” (eg, shortness of breath, bowel problems, cough, and weakness) or scheduled health care use. This lack of effects on physical outcomes is consistent with the weak mostly null effects yielded in a meta-analysis of 16 RCTs of in-person primary care interventions for multimorbidity [22] and is in contrast to the moderate effects on lab scores observed in a meta-analysis of digital telemedicine interventions [25], perhaps reflecting the fact that the latter interventions tended to engage medical care more directly and specifically (eg, via online tracking of blood pressure). On the other hand, those interventions found scant effects on patients’ QOL.

Our expanded inclusion criteria meant that participants could have varied combinations of 11 chronic conditions. This was arguably a strength of the study, in that such diverse arrays are a key challenge of treating patients with multimorbid conditions. On the other hand, this design decision may also have contributed to the lack of effects on physical outcomes and the relatively weak effects on mental QOL, in that some patients’ chronic conditions need not have been particularly onerous. A patient could have high blood pressure, high cholesterol, and a BMI over 30 without feeling much incentive to engage with the health-related aspects of the intervention and without much impact on their ability to engage in social activities. Although, as shown in Multimedia Appendix 1, the majority of participants in both study arms had EHR diagnoses of chronic pain or arthritis, there were signs that patients did not see themselves as incapacitated and that many had active social lives. In exit interviews, participants made comments such as, “If I had more medical problems, this could be more helpful. But I am pretty healthy” and “I don't have any chronic aches or pains, nothing constant that bothers me. I think this doesn't apply to me.” Others made comments about their high levels of social engagement as a reason for not needing the interactions offered by ET (eg, “I'm not a shut-in. We're very active, do lots of things with family, don't need interaction with other people that much”). In our current RCT of a subsequent iteration of ET, we are focused on older adults with at least 5 (rather than 3) chronic conditions to probe whether there are indeed stronger effects for patients with more complex sets of conditions.

It is also possible that the effects of ET were reduced by the overlap of the study with the pandemic. A key component of ET was the health tracking survey and the ensuing clinician report. The goals of the clinician report were to not only flag potential issues for health care providers (eg, recent falls could signal important health risks) but also open conversations during appointments about issues, such as sleep, diet, or mood, that may not be primary concerns for clinicians in the context of more urgent medical indicators but that may affect patients’ physical and socioemotional QOL. As clinic visits ceased and clinical staff were triaging the crises of the pandemic, the potential for such expansive conversations decreased, even though we adapted to send clinics a summary of their patients’ health tracker responses. However, despite the intuitive appeal of the pandemic as a potential limiting factor, we found no indication that days of overlap with the pandemic moderated the effects of the study arm for either physical or socioemotional outcomes. Approximately half of the sample who completed the 12-month intervention period prior to the pandemic did not appear to respond differently than those who overlapped with the pandemic.

It is also worth noting that these analyses leave the pattern of stronger effects for women (vs men) unexplained. One possibility is that there were sex differences in the types of messages posted and received on ET and that this had implications for participants’ socioemotional outcomes. A 2020 meta-analysis found that women were more likely than men to give social support in online communities (eg, social networking sites) and were also slightly more likely than men to receive social support [77]. This pattern of sex differences was also observed in a CHESS intervention for alcohol use disorder [78]. Moreover, giving and receiving social support via the discussion groups in CHESS interventions was previously found to predict more positive outcomes: improved mental well-being in a CHESS intervention for women with breast cancer [79] and reduced drinking in a CHESS intervention for alcohol use disorder [80]. Further work is needed with the current data set to code the types of online interactions that took place on ET and to assess how they may have contributed to socioemotional outcomes.

### Limitations

We have already alluded to several limitations, including the issues of multiple primary outcomes potentially inflating type 1 error and the lack of EHR lab data. The latter issue means that this is a study of the effects of ET on older adults’ perceptions of their physical status, rather than a study of whether the intervention could induce sufficient behavioral changes to be reflected in participants’ physical health indicators. While our focus was more on participants’ QOL than lab scores, as evidenced by our choice of primary versus secondary outcomes, this nonetheless leaves an important question about physical effects to be answered by further work.

As noted in the Methods section, we lost the 18-month follow-up timepoint, given our ethics-based decision to allow participants during the pandemic to continue their use of ET beyond the 12-month study period. The goal of the follow-up period is, of course, to assess whether any effects observed during the intervention period endure even after the intervention ends. While we acknowledge the loss of these insights, we also note that they may be less critical than for other types of interventions. Unlike interventions that are highly burdensome for clinicians or that involve biomedical procedures, the online accessibility of ET resources could potentially be an ongoing open-ended opportunity for patients, rather than coming to an end once a “dose” is completed.

Perhaps a more substantive limitation was the lack of data about the clinician report. We originally intended to gather both quantitative and qualitative data from primary care clinicians to gain insights into the ways they did (or did not) engage with patients’ health tracking data. Given the effort involved in making sure that clinicians received the relevant report in a timely manner before a patient’s appointment, it would have been useful to understand the results of those efforts. This was intended to be an intervention implemented within primary care. It is possible that the benefits could be observed even in the absence of engaging directly with clinics, simply by encouraging participants to reflect on their health tracking data and to use their EHRs or clinic visits to flag changes over time that mattered to them. The relatively high sustained rates of engagement with the tracker suggested that participants found some utility, but we did not obtain their ratings of the helpfulness of the tracker or the associated clinician report. Measures of patient perceptions of the intervention or the 6- and 12-month participant surveys would have helped us assess the value of the feature and determine possible improvements for future interventions.

Finally, it is important to highlight that participants were all located in Wisconsin and were mainly white (92.2%), with at least some education beyond high school (78.8%), limiting the generalizability of the results. This limitation reflected our focus on implementing ET into primary care and our goal of using EHR data to assess physical outcomes. The complexity of engaging with clinics and accessing EHR data limited the number of clinics that participated and restricted us to 1 health care organization. A possible solution is to prioritize the accessibility of ET over the as-yet-unknown benefits of trying to implement ET within specific primary care clinics. In our ongoing RCT, we have made that trade-off by recruiting participants via television and social media rather than through specific clinics, allowing us to recruit a more diverse sample.

### Conclusions

This study extends our prior findings by focusing exclusively on the growing complex population of older adults coping with MCCs. The current findings continue to support the value of ET for patients’ socioemotional outcomes, with less impact on physical outcomes. Subsequent RCTs of ET are underway with (1) older adults experiencing chronic pain and (2) older adults with 5 or more chronic conditions for whom there may indeed be greater decrements to physical and mental QOL, with content more explicitly focused on addressing their serious health-related needs.

## Appendix

Multimedia Appendix 1 Additional prespecified analyses: secondary and exploratory outcomes.

Multimedia Appendix 2 ElderTree system samples.

Multimedia Appendix 3 CONSORT-eHEALTH checklist (V 1.6.1).

Multimedia Appendix 4 Breakdown of chronic conditions by group.

## Abbreviations

CHESS: Comprehensive Health Enhancement Support System
EHR: electronic health record
ET: ElderTree
MCCs: multiple chronic conditions
QOL: quality of life
RCT: randomized controlled trial
UC: usual care
UC+ET: usual care+ElderTree access (experimental condition)
UC+internet: usual care+internet access but no ElderTree (attention control condition)
