# Supplementary material for: An eHealth Intervention to Improve Quality of Life, Socioemotional, and Health-Related Measures Among Older Adults With Multiple Chronic Conditions: Randomized Controlled Trial
Source: JMIR Aging. 2024 Dec 6;7:e59588. doi: 10.2196/59588 (PMC11662192; doi:10.2196/59588)
Supplement: Multimedia Appendix 1 [file aging_v7i1e59588_app1.docx]

**Multimedia Appendix 1.** Additional prespecified analyses: secondary and exploratory outcomes.

**Secondary Outcomes**

***Methods***

Secondary outcomes, intended to assess a wide range of physical health indicators, were number and severity of falls, symptom distress, medication adherence, crisis healthcare use, long-term care use, and health outcomes from lab scores.

Falls, defined in the survey as "the body going to the ground without being pushed," were assessed with 2 items: how many times the participant fell in the past 3 months and how many falls required medical attention.

Symptom distress was assessed using a combined list of 20 symptoms from the General Symptom Distress Scale [1] and Bayliss’s Disease Burden Scale [2] rating severity for each item (eg, pain, weakness, cough, skin rashes or sores) on a 6-point scale. Total possible score range was 0–120, with higher scores signifying greater distress.

Medication adherence was measured with eight 5-point items. For 6 items from the Brief Medication Questionnaire,[3] participants rated how often they had specific issues with medication (eg, "I forget sometimes," "It is hard to pay for my medication"). Based on experiences with patients, we added 2 original items: “Feels like I no longer need it" and "Feels like I don’t need the full dose.” The total possible range was 8–40, with higher scores signifying greater challenges or inconsistency with medication adherence.

For crisis healthcare use, participants were asked to report, for the preceding 3 months, their number of urgent care visits, emergency room visits, and hospitalizations, plus number of days of each hospitalization, with the latter 2 items used to calculate number of 30-day hospital readmissions. Participants also reported long-term care use (number of nights spent in assisted living facilities and nursing homes) over the past 3 months.

We originally planned to test multiple health outcomes from lab scores (mmHg for hypertension, mg/dL for hyperlipidemia, HbA1c for diabetes, body mass index, and Visual Analogue Scale [4] pain ratings) but were unable to obtain sufficient data from electronic health records. Therefore, we could not test these variables, with the exception of pain, which we instead assessed via a single survey item from the PROMIS, "How would you rate your pain on average?" The ranking range was 0–10, with 0 signifying "no pain" and 10 signifying "worst imaginable pain."

***Results***

The ElderTree arm showed a slight increase in pain over time while the control arm showed a slight decrease (b=0.25, CI 0.03 to 0.47, *P*=.028). The full range of pain scores, 0–10, was observed among participants. See Figure S1 for the model-estimated means over time and a visual representation of the interaction. By 12 months, the difference in means was a statistically significant but small effect (12-month mean difference=0.49, *P*=.045, Cohen’s d=0.22). We are presently conducting a randomized controlled trial that examines the effects of a modified ET on chronic pain in the context of multimorbidity.

**Figure S1.** Predicted mean values of the PROMIS pain scale over time.


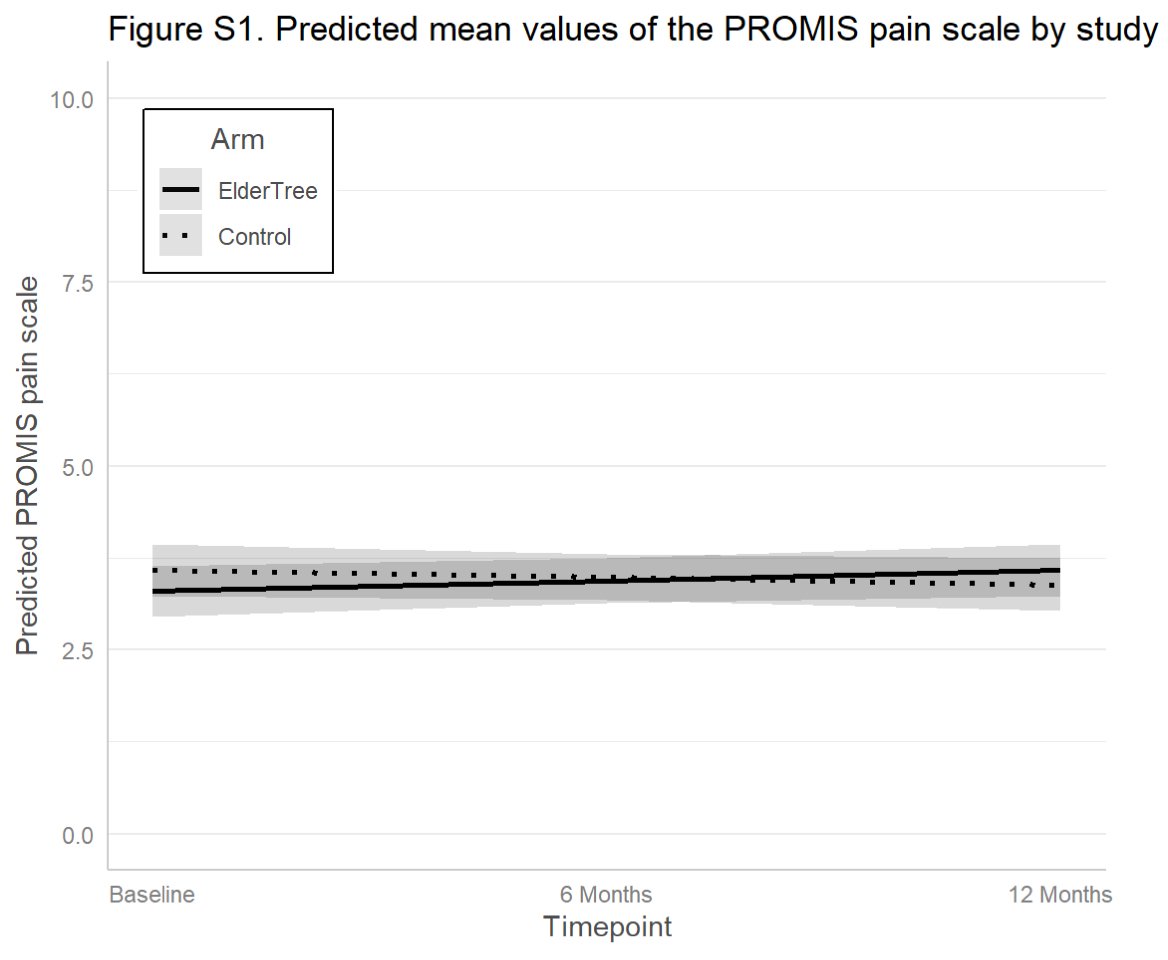


Note. Possible range is 0–10, with higher values indicating more pain. Shaded areas are 95% confidence intervals.

We did not find significant effects of ET for falls, symptom distress, medication adherence, or use of crisis healthcare. Table S1 shows inferential statistics for all secondary outcomes except number of falls requiring medical attention and use of long-term care, for which there was too little data for testing.

**Table S1.** Inferential statistics for secondary outcomes.

| **Outcome** | **Inferential statistics for Arm x Timepoint** | | |
| --- | --- | --- | --- |
|  | **Estimate** | **95% Cl** | ***P* value** |
| Pain (PROMIS) | 0.25 | 0.03 to 0.47 | .028 |
| Medication adherence | –0.02 | –0.43 to 0.39 | .94 |
|  | **OR** | **95% Cl** | ***P* value** |
| Any falls (yes/no)^a^ | 1.00 | 0.63 to 1.59 | .99 |
| Any crisis healthcare use (yes/no)^a^ | 0.97 | 0.65 to 1.47 | .90 |
|  | **Incidence rate ratios** | **95% Cl** | ***P* value** |
| Symptom distress | 1.02 | 0.96 to 1.09 | .45 |

^a^Outcome was analyzed as yes/no because there was not enough variance to test by type or number of event.

#### **Exploratory Outcomes**

***Methods***

Exploratory outcomes included behavioral variables that may impact a number of chronic conditions. Participants rated 7 dietary items on a 5-point scale, indicating how often they ate various healthy and unhealthy foods. The possible score range was 7–35, with higher scores equating with less healthy habits. Participants also reported problem drinking with items 3–10 of the Alcohol Use Disorders Identification Test (AUDIT).[5] The total possible score range was 0–30, with higher scores indicating more alcohol use. A count of pain medication issues was taken using a modified 8-item list from the CDC (Centers for Disease Control and Prevention) Pain Medication Survey (eg, "My insurance has refused to pay for my pain medication"); counts ranged 0–7.[6] Participants also reported the number of cigarettes smoked per day.

***Results***

We did not find effects of study arm on changes in dietary habits, pain medication issues, or alcohol use problems at any time point, and we were unable to test cigarette use due to a lack of participants who smoked during the study (n=17). (For inferential statistics, see Table S2.)

**Table S2.** Inferential statistics for exploratory outcomes.

| **Outcome** | **Inferential statistics for Arm x Timepoint** | | |
| --- | --- | --- | --- |
|  | **OR** | **95% Cl** | ***P* value** |
| Alcohol use problems (yes/ no) | 1.13 | 0.46 to 2.81 | .78 |
| Pain medication issues | 0.64 | 0.27 to 1.55 | .33 |
|  | **b** | **95% Cl** | ***P* value** |
| Diet | –0.20 | –0.52 to 0.11 | .21 |

**Additional Exploratory Analyses: Post-Hoc Probing of Moderation by Sex**

Given that the effect of study arm on mental quality of life was mediated by interim improvements in relatedness, we examined whether this indirect path was moderated by sex: perhaps the impact of ET on relatedness primarily occurred for women and this would explain why women showed stronger effects of ET on mental quality of life and psychological well-being. In fact, sex did not interact with study arm to predict 6-month increases in relatedness (b= –1.19, CI –3.67 to 1.29, *P*=.35). Thus, relatedness does not explain the stronger effects of ET for female participants. It is worth noting, however, that these analyses found that 6-month improvements in relatedness predicted 12-month improvements in psychological well-being.

**References**

1. Badger TA, Segrin C, Meek P. Development and validation of an instrument for rapidly assessing symptoms: The general symptom distress scale. J Pain Symptom Manage. 2011;41(3):535-548. PMID:21131168

2. Bayliss EA, Ellis JL, Steiner JF. Seniors’ self-reported multimorbidity captured biopsychosocial factors not incorporated into two other data-based morbidity measures. J Clin Epidemiol. 2009;62(5):550-557.e1. PMID:**18757178**

3. Svarstad BL, Chewning BA, Sleath BL, Claesson C. The Brief Medication Questionnaire: A tool for screening patient adherence and barriers to adherence. Patient Educ Couns. 1999;37(2):113-124. PMID:14528539

4. Crichton N. Information point: Visual Analogue Scale (VAS). J Clin Nurs. 2001;10(5):706. In Gould D, Kelly D, Goldstone L, Gammon J. Examining the validity of pressure ulcer risk assessment scales: Developing and using illustrated patient simulations to collect the data. J Clin Nurs. 2001;10(5):697-706. PMID:**11822520**

5. Saunders JB, Aasland OG, Babor TF, de la Fuente JR, Grant M. Development of the Alcohol Use Disorders Identification Test (AUDIT): WHO Collaborative Project on Early Detection of Persons with Harmful Alcohol Consumption--II. Addiction. 1993;88(6):791-804. PMID**:8329970**

6. Pain News Network. 2017 CDC survey results. Accessed July 17, 2023. https://www.painnewsnetwork.org/2017-cdc-survey/
