# Supplementary material for: An eHealth Intervention to Improve Quality of Life, Socioemotional, and Health-Related Measures Among Older Adults With Multiple Chronic Conditions: Randomized Controlled Trial
Source: JMIR Aging. 2024 Dec 6;7:e59588. doi: 10.2196/59588 (PMC11662192; doi:10.2196/59588)
Supplement: Multimedia Appendix 2 [file aging_v7i1e59588_app2.docx]

**Multimedia Appendix 2.** ElderTree system samples.

**Figure S1.** ElderTree home screen.

**
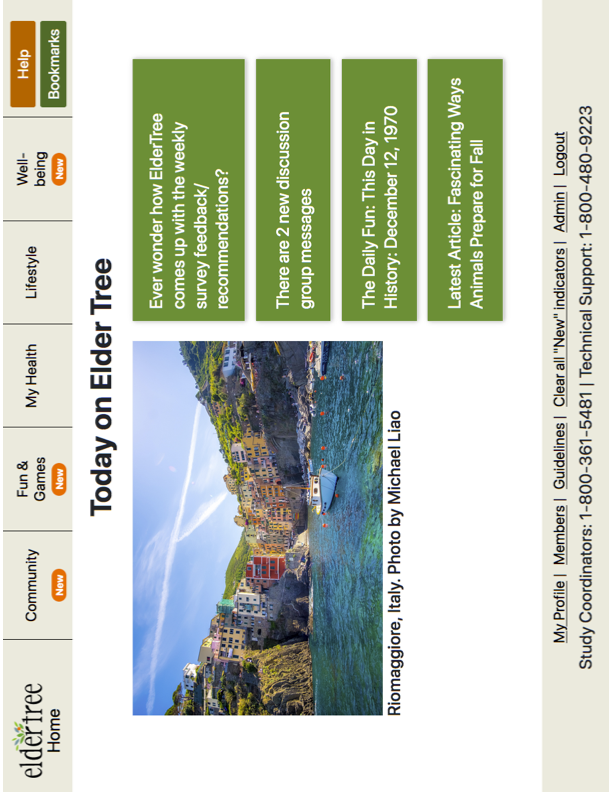
**

**Figure S2.** ElderTree clinician report.

**Figure S3.** ElderTree clinician report summary.
