# Supplementary material for: An eHealth Intervention to Improve Quality of Life, Socioemotional, and Health-Related Measures Among Older Adults With Multiple Chronic Conditions: Randomized Controlled Trial
Source: JMIR Aging. 2024 Dec 6;7:e59588. doi: 10.2196/59588 (PMC11662192; doi:10.2196/59588)
Supplement: Multimedia Appendix 4 [file aging_v7i1e59588_app4.docx]

**Multimedia Appendix 3.** Breakdown of chronic conditions by group.

**Table S1.** Number (%) of patients with each number of chronic condition diagnoses at baseline.

| **Number of chronic condition diagnoses in patient's electronic health record** | **Control**  **(N=168)** | **ElderTree**  **(N=176)** |
| --- | --- | --- |
| 3 conditions diagnosed | 9 | 10 |
| 4 conditions diagnosed | 40 | 43 |
| 5 conditions diagnosed | 55 | 55 |
| 6 conditions diagnosed | 41 | 33 |
| 7 conditions diagnosed | 14 | 25 |
| 8 conditions diagnosed | 7 | 7 |
| 9 conditions diagnosed | 1 | 2 |
| 10 conditions diagnosed | 1 | 1 |
| 11 conditions diagnosed | 0 | 0 |

**Table S2.** Number of patients diagnosed with each of 11 chronic conditions at baseline.

| **Diagnosis in electronic health record** | **Control**  **(N=168)** | **ElderTree**  **(N=176)** |
| --- | --- | --- |
| Arrhythmia/atrial fibrillation | 24 | 39 |
| Arthritis | 100 | 101 |
| Body mass index (BMI) ≥30 | 118 | 114 |
| Chronic kidney disease | 71 | 78 |
| Chronic obstructive pulmonary disease (COPD) | 23 | 19 |
| Chronic pain | 114 | 120 |
| Congestive heart failure | 10 | 19 |
| Diabetes | 104 | 112 |
| Hyperlipidemia | 143 | 149 |
| Hypertension | 164 | 173 |
| Pulmonary heart disease or pulmonary vascular disease | 8 | 10 |
